# Ten Tips for Engaging the Millennial Learner and Moving an Emergency Medicine Residency Curriculum into the 21st Century

**DOI:** 10.5811/westjem.2016.3.29863

**Published:** 2016-05-05

**Authors:** Shannon L. Toohey, Alisa Wray, Warren Wiechmann, Michelle Lin, Megan Boysen-Osborn

**Affiliations:** * University of California Irvine, Department of Emergency Medicine, Orange, California; † University of California San Francisco, Department of Emergency Medicine, San Francisco, California

## Abstract

**Introduction:**

Millennial learners are changing the face of residency education because they place emphasis on technology with new styles and means of learning. While research on the most effective way to teach the millennial learner is lacking, programs should consider incorporating educational theories and multimedia design principles to update the curriculum for these new learners. The purpose of the study is to discuss strategies for updating an emergency medicine (EM) residency program’s curriculum to accommodate the modern learner.

**Discussion:**

These 10 tips provide detailed examples and approaches to incorporate technology and learning theories into an EM curriculum to potentially enhance learning and engagement by residents.

**Conclusion:**

While it is unclear whether technologies actually promote or enhance learning, millennials use these technologies. Identifying best practice, grounded by theory and active learning principles, may help learners receive quality, high-yield education. Future studies will need to evaluate the efficacy of these techniques to fully delineate best practices.

## INTRODUCTION

Medical education is rapidly changing as millennial learners place priority on self-directed learning, using a digital library of videos, podcasts, social media, and other online resources.^1^–^3^ The status quo of routine, classroom lectures does not meet millennial learner needs. We propose that a learner-centered approach with active engagement optimizes knowledge retention.

While research on effective teaching strategies in graduate medical education is still lacking, many aspects of educational theory are well established. These 10 tips for engaging the millennial learner and moving an emergency medicine (EM) residency into the 21st century incorporate these principles and discuss working examples to advance one’s residency curriculum to better engage today’s learners.

### Tip #1: “Flip” your curriculum

While textbooks and traditional references provide residents with a foundation of basic concepts and knowledge,^4^,^5^ classroom time should avoid regurgitating textbook facts and figures. Instead, these didactic sessions should focus on clarifying points of confusion, explaining high-order concepts, or challenging learners to apply concepts to clinical scenarios.^6^,^7^ A “flipped classroom” requires residents to complete basic learning at home. Then, they attend didactic sessions that promote active learning. This model ensures that residents are taught both basic and advanced concepts, while remaining engaged. Studies on the “flipped classroom” model in undergraduate and graduate health professions education have been small, but have demonstrated improved learner satisfaction and either improved or similar knowledge acquisition when compared to traditional methods of instruction.^8^–^16^ We see an additional benefit of the flipped classroom in promoting lifelong learning, but this has not been studied.

Flipping the residency program curriculum is multifaceted and incorporates many existing curricular practices. Residency programs must first outline the content they expect their residents to complete asynchronously. We call this learner responsible content (LRC). Traditionally, programs recommend a textbook reading schedule; however, as digital media such as blogs, podcasts, and reference websites permeate the Internet^17^ and become increasingly popular with EM learners,^18^,^19^ these sources should be included in the LRC. Because some of these digital resources are not written by experts, peer-reviewed, or consistently reliable,^20^ it is imperative that programs guide their learners to dependable sources or use quality indicators to evaluate educational blogs and podcasts.^21^,^22^ Life In The Fast Lane Review and the Academic Life in Emergency Medicine (ALiEM) Approved Instructional Resources (AIR) Series are resources that curate high-quality content.^22^,^23^

Once the curriculum of LRC is established, in-classroom didactics should then build on the foundation of LRC through such application exercises as problem-based learning (PBL), audience response systems (ARS), and team-based learning (TBL). This allows residents to pursue higher order Bloom taxonomy levels^24^ of learning. By aligning didactic content with LRC, the learner has the impetus to complete the assignments and may benefit from improved knowledge retention, as material is repeated, built upon, and applied.^25^

### Tip #2: Incorporate individualized interactive instruction into a learning management system

The flipped curriculum blends nicely with asynchronous learning. In 2008, the Accreditation Council for Graduate Medical Education’s (ACGME) EM Residency Review Committee (RRC) and Council of Residency Directors (CORD) recommended that a program’s ideal didactic curriculum should include a mixture of asynchronous and synchronous learning.^7^ The EM RRC began allowing up to 20% of the didactic curriculum to be replaced by asynchronous learning, termed individualized interactive instruction (III)*.* Resident participation in III requires that learning be monitored by the program director, evaluated, overseen by faculty, and monitored for efficacy.^26^,^27^ A learning management system (LMS), such as Schoology, Canvas, or BlackBoard, can organize a program’s LRC, providing a platform for learners to access a menu of videos, interactive educational modules, articles, quizzes, recorded didactics, and other assignments. Furthermore, an LMS can help meet the above four III criteria. Costs for implementing an LMS at a residency program can vary from free (Schoology) to a per-learner cost (Blackboard, Canvas) that may be covered by a university’s contract. The benefits of III include identifying and addressing an individual learner’s needs; encouraging practice-based learning and improvement and lifelong learning; and the ability to use expert content for asynchronous materials.^7^

### Tip #3: Incorporate modern didactic approaches

Small group learning (SGL) is an ideal format for high-yield, interactive didactic sessions. Its benefits include providing opportunities for assessment, clarification of knowledge gaps, improved learner engagement, teamwork, and a varied didactic format. Anecdotally, SGL uses more resources than are feasible for routine use; however, in our experience it requires similar preparation to a standard, high-quality lecture. Between faculty, fellows, local alumni, and chief residents, programs should have a sufficient number of instructors. Instructors should have clear objectives for the learning sessions, as well as information on how to best facilitate the session. The ideal SGL session is self-directed by learners, with faculty available to provide additional information, clarify any points of confusion, ask open-ended questions, and ensure the group remains on track.^28^–^32^

Small group learning sessions can be carried out through a TBL or PBL approach with application exercises or may focus on areas such as board review or visual diagnosis. While instructors can create content de novo, there are many sources to acquire existing learning materials, such as MedEdPORTAL,^33^ CORD Teaching Cases,^34^ as well as many other textbook and Internet sources. In undergraduate medical education, PBL has been shown to be more effective than standard instruction in improving clinical performance, knowledge and reasoning,^35^ while TBL showed improvement of examination scores.^36^ Its efficacy has not been as well studied in graduate medical education.

Problem-based learning: A group works through a clinical vignette in a small group setting,^37^ encouraging self-directed learning and self-evaluation. Facilitators are available as expert “consultants” to answer questions.^38^

Team-based learning: Previous research suggests that millennial learners prefer team-oriented projects and learning.^2^ Team-based learning exercises require only a single instructor. After completing LRC, learners participate in an individual readiness assurance test (iRAT), followed by a group readiness assurance test (gRAT), ensuring learners “know” the content.^39^ Groups then apply their knowledge during a group application exercise where the learners “show how”.^39^ A facilitator reviews learning points and clarifies any confusion. Team-based learning encourages teamwork and communication, improves learning outcomes and examination scores, and develops lifelong learning skills.^6^

### Tip #4: Improve lecture efficacy by keeping it high yield and brief

While lectures have a negative reputation in modern learning theory, they should not be eliminated entirely from residency didactics. Lectures are practical, as they require only one instructor for an almost unlimited number of learners. However, rather than regurgitate textbook information, lecturers should use the time to build on concepts that have been covered in textbooks or other LRC.

Because experts suggest that learners only absorb 5% of lecture material and have an attention span of only 15–20 minutes,^40^,^41^ decreased lecture time, coupled with methods that promote active learning (pause procedures, simulation, small group discussion), should improve retention.^6^ For example, our residency program frequently divides the 45-minute “lectures” into a 15-minute lecture, followed by a 30-minute SGL exercise. Alternatively, a short lecture, clarifying commonly asked questions or reiterating concepts, could follow a TBL exercise. Instructors can better engage learners through commitment activities, such as audience response software (ARS) and pause procedures, such as the “one-minute paper” approach.^6^ Wolff and colleagues provide an outstanding overview of multiple methods for engaging learners in didactic lectures (Table).^6^ Further methods that ensure high-quality lectures include using a manageable scope of content, clear objectives, and case-based scenarios.^42^

Not all topics, however, are best taught using SGL. For instance, more dense material can be covered in a required reading or multimedia assignment, followed by a high-yield board review lecture with ARS or a game format, both of which have been shown to increase engagement, motivation, and retention.^43^–^49^

### Tip #5: Have a central, cohesive technology plan for resident education

Establishing a central agreed-upon technology, such as iPads or other tablet devices, can be essential to ensuring consistent access to online resources and electronic textbooks.^50^ The tablet provides access to the program’s LMS, which provides an organized, real-time updated database of the LRC and monitoring of modules, projects, and quizzes. Mobile access to LRC can encourage completion of assigned reading by providing more convenient, portable access.^51^ Our residency program uses Schoology, a free LMS, which is functionally advanced and user friendly for both administrators and learners.

### Tip #6: Use simulation to its full potential

Simulation, broadly defined, includes a number of interactive tools, such as human simulation with “standardized patients,” oral board cases, games, computer-based training exercises, or the more realistic high fidelity, mannequin-based simulators. Simulation provides team-based, engaging, relevant, active learning with emotional connection. It is an ideal supplement for residency curriculum because it allows educators to expose residents to uncommon or critical situations in a more realistic form.^52^,^53^ Furthermore, simulation exercises can be another tool in the flipped classroom model for residents to demonstrate and solidify lessons from their LRC.

Simulation has been shown to be an effective teaching strategy, with studies demonstrating improved quality of care, knowledge retention, comfort in performing procedures, and performance in repeated simulated medical scenarios.^54^,^55^

While the initial investment of a simulation center can be quite high, once the physical space and resources exist, the creation of cases and content can be developed by faculty and residents, or found in any number of online databases. Sharing a simulation center with other departments or local institutions may be an option to help finance it.

### Tip #7: Think of any moment as a potential teaching moment

Traditionally, teaching is restricted to on-shift (clinical) learning and in-classroom didactics, with LRC serving as supplemental learning material. With modern technology (smartphones, tablets, and laptops), educators have the opportunity to create educational opportunities throughout the day beyond these traditional teaching venues.

For example, web services can deliver pre-scheduled or automated announcements via email,^56^ text messages, tweets, or Facebook posts. These might include images, key facts, or even brief quizzes that residents can access while on the go. Tablets that display board review questions or visual diagnosis quizzes can be placed in charting rooms (i.e., a “fact board”), so that learners can engage in a steady stream of information throughout their shift, although we have found that medical students, nurses, and technicians seemed to engage in the fact board more often than our residents. Publishing periodic educational content on a residency-run blog, which can be likened to an online magazine or journal, can be used to post interesting cases, or discuss diseases or local relevant medical news. These resources should be updated frequently so that residents consistently have the most useful information available.

### Tip #8: Use technology for more effective, formative feedback

Traditional feedback exists via Likert scale-based competency assessment, with comment boxes for formative feedback. The implementation of the ACGME Milestones was intended to provide a more consistent assessment process, to accurately describe a residents’ performance and allow tracking of improvement.^26^,^57^ However, it still provides rote, non-personalized, summative feedback that does not necessarily help the resident improve specific skills. Furthermore, a traditional semi-annual format of evaluations may diminish faculty recall of resident performance. Millennial learners value prompt, “real-time” feedback.^2^ Evaluation apps and programs can be used to encourage more frequent assessment, which may lead to real-time feedback and a more accurate assessment of a resident’s overall performance. Real-time feedback may result in more formative feedback, which can drive further learning.^58^ We use Instant Eval, a program built for milestone assessment during or after a shift. (Other options include New Innovations and MyEvaluations.com.)

It can be especially challenging for supervisors to provide feedback to learners during procedures and resuscitations. Providing specific, real-time, formative feedback can be difficult for faculty, as their attention is often split between managing the patient and observing the resident. Faculty members also may be hesitant to verbalize feedback in front of patients in order to preserve confidence in their providers. Furthermore, busy EM faculty may not observe the entirety of a procedure, missing subtle technical errors. Because of these reasons, procedural feedback is typically provided afterwards, often resulting in recall bias and focusing on the success or failure of the procedure as the major outcome. Meanwhile technical difficulty and other factors are forgotten. One potential solution is first-person video recording using optical head-mounted displays, such as Google Glass or Go Pro cameras. These devices can capture the entire procedure and are non-obstructive to patient care. Faculty can review these recordings, and annotate them using an application such as Coach’s Eye or notes with associated time- stamp marks. This allows more formative, substantial evaluation which could lead to resident improvement on procedural skills,^59^,^60^ communication during resuscitations, and general approach to an acutely ill patient. While only a few EM residency programs use video recordings for feedback, these programs find that it the most effective method for providing feedback to residents.^61^

First-person recording has associated HIPAA compliance issues, as many of the patients undergoing difficult procedures and resuscitations cannot consent to recording. Many programs already record traumas and resuscitations for quality improvement, and this first-person recording can be used in the same way. However, it will require approval from hospital compliance committees, as well as appropriate software to ensure encrypted recordings.

### Tip #9: Teach educators to be proficient in a variety of educational technologies through professional development opportunities

Educators should be proficient in a variety of educational approaches both involving and not involving technologies in order to best accomplish particular educational objectives.^26^ Professional development sessions can help faculty members use effective bedside teaching and lecturing, audience response systems, and small group learning to enhance resident learning experiences.^62^ Active experimentation and application of various techniques in these sessions allows educators to experience, troubleshoot, and observe the operational details.

The culture of technology can be further promoted through educational fellowship programs. The University of California, Irvine is the first to offer a fellowship in Multimedia Design and Educational Technologies (MDEdTech). This program offers fellows the opportunity to participate in residency curriculum design, creation and curation of educational technologies for residents and medical students, and the use of educational technologies for patient education.

### Tip #10: Identify an instructional technology champion in the department

With the ongoing growth and evolution of new instructional technologies and digital resources, it can be challenging for any residency program to stay current and abreast of these developments. A program should identify a faculty or resident (such as a chief resident of technology) to monitor the medical education literature, educational technology websites (e.g. EducatorsTechnology.com, Edudemic.com, Edutopic.org), technology websites (e.g. TechCrunch.com, iMedicalApps.com), and key Twitter accounts, (e.g. @Educ_Technology and @EduTweetTech). Furthermore, s/he could periodically check for new blogs or podcasts that demonstrate high educational value for EM residents to incorporate into the LRC curriculum.

### Effectively applying these 10 tips to your residency

In our own residency program, we have incorporated each of these “tips” over the past three years. In the first phase of these curricular modifications, we replaced one hour of conference time with asynchronous curricula. Despite decreasing conference time, we saw no associated change of in-training exam scores pre- and post-implementation.^63^ While it is reassuring that in-training exam scores did not decrease, we felt that we were not properly organizing and integrating this asynchronous curriculum into our synchronous curriculum and we were not meeting the requirements of III. Now, we have better aligned our asynchronous and synchronous curriculum and added back 30 minutes of conference (total conference time 4.5 hours) in order to review asynchronous content. Our chief resident of technology works with our residency leadership team to identify, create, and post III content onto our LMS.

In the second phase of our curricular restructuring, we revised our didactic (synchronous) curriculum. To implement these changes, we formed a curriculum planning committee (CPC), which includes the program director, assistant program directors, and MDEdTech fellows. We held a two-hour faculty development session to introduce TBL, SGL, and other effective didactic methods to our faculty members. We reminded faculty members about these concepts at monthly faculty meetings and educated our chief residents during a “chief dinner” at the beginning of the academic year. We assigned a faculty member, academic chief resident, and member of the CPC to each block of didactic curriculum. The faculty member and chief resident do the majority of planning for the conference block, but the CPC member ensures that the appropriate content is covered and that the didactic curriculum incorporates effective learning strategies.

Time- and/or feedback-based modifications have helped to overcome many of the challenges we have faced in implementing these curricular changes. For example, we found that senior level residents had the lowest participation in our asynchronous curriculum when we first implemented it. Now, since our current senior residents have used our LMS since the start of their residency, we have equal participation between the senior and junior learners in III. We have an 80% completion rate among all classes.

The chief residents have been integral to ensuring the success of this culture change. They have encouraged faculty members of all experience levels to incorporate these strategies into their didactics and have helped create or find much of the content for our SGL exercises.

## CONCLUSION

Education is changing rapidly with the current instructional technologies of the 21st century, and medical education is no different. Although it is still unclear whether these technologies are actually more effective than previous, less technologically focused approaches, millennial learners are using social media and other technologies for their learning. Residencies will be well served by becoming proficient in these digital tools in order to provide an optimal resident learning experience. When any new digital innovation or technology is incorporated in the curriculum, it should be evaluated in terms of efficacy, logistical burden, accuracy, and user experience. These experiences, whether positive or negative, should be shared with others in a public forum to help the greater education community more quickly identify best practices in modern medical education.

## Figures and Tables

**Table:** Techniques to engage learners in didactive lectures.*

Technique	Description
Commitment activities	Learners are posed a question and must “commit” to a response. Examples: audience response system, iRATs, multiple choice questions, visual diagnosis.
Pause procedures	A brief “pause” in a didactic session to allow learners to clarify and assimilate learning points. Examples: One-minute paper where learners spend one-minute writing a response to a question posed by the instructor (also a commitment activity) and the Muddiest Point where learners reflect on and share on primary points of confusion
Jigsaw	Learners are tasked to become an expert on one of many small parts of a particular topic. Each expert teaches his part of the topic to the other learners. In the end, the topics all come together like a jigsaw puzzle.
Role-play	Specific to case scenarios, learners play a part (for example patient, physician or family) to work through a case and understand concepts and theories.
Think-pair-share	Instructors ask learners a question. Learners are encouraged to “think” about their response. Then, they “pair” with a neighbor and “share” their answers with each other.

*iRAT*, individual readiness assurance test

* Adapted from reference 6.
